# Knowledge embedded

**DOI:** 10.1007/s11229-019-02326-2

**Published:** 2019-07-31

**Authors:** Dirk Kindermann

**Affiliations:** Institute of Philosophy, Universität Wien, Universitätsring 1, 1010 Vienna, Austria

**Keywords:** Knowledge, Context-sensitivity, Pragmatic invariantism, Implicatures, Embedded implicatures, Epistemic contextualism

## Abstract

How should we account for the contextual variability of knowledge claims? Many philosophers favour an invariantist account on which such contextual variability is due entirely to pragmatic factors, leaving no interesting context-sensitivity in the semantic meaning of ‘know that.’ I reject this invariantist division of labor by arguing that pragmatic invariantists have no principled account of embedded occurrences of ‘*S* knows/doesn’t know that *p*’: Occurrences embedded within larger linguistic constructions such as conditional sentences, attitude verbs, expressions of probability, comparatives, and many others, I argue, give rise to a threefold problem of embedded implicatures.

## Introduction

It is often the case that we can use the same words to communicate different things in different contexts. The word ‘know’ is no exception. Epistemologists and philosophers of language have spent a good deal of their time and effort trying to find out the ways in which what we communicate, when we assert declarative sentences of roughly the form ‘*S* knows that *p*’ and ‘*S* doesn’t know that *p*’, depends on features of the context; which features these are; and which contexts matter.

So how should we account for the contextual variability of knowledge claims? In the dispute over the meaning of the word ‘know’, parties on all sides have favoured a simple methodology to test for contextual variation. The methodology can be called ‘simple’ because it focuses on assertions of simple, or unembedded, knowledge sentences—sentences of roughly the form ‘*S* knows that *p*’ and ‘*S* doesn’t know that *p*.’ Such sentences are presented together with one case of a minimal-contrast pair, whose cases differ only in some practical and/or psychological feature; speakers are subsequently tested for their intuitive judgments of the sentence’s truth or felicity.

Early debates prompted by the advent of contextualism as well as recent experimental studies concerned with ‘know”s pattern of contextual variation have worked almost exclusively from simple knowledge sentences. This isn’t surprising. Keith DeRose, in championing the contextualist’s case for ‘know”s variability, advertises a ‘methodology of the straightforward’:

A certain methodology would strongly favour contextualism. This ‘methodology of the straightforward’, as we may call it, takes very seriously the simple positive and negative claims speakers make utilizing the piece of language being studied, and puts a very high priority on making those natural and appropriate straightforward uses come out true, at least when that use is not based on some false belief the speaker has about some underlying matter of fact. Relatively little emphasis is then put on somewhat more complex matters...(DeRose 2009, p. 153)

There is much to applaud in DeRose’s statement on methodology. However, the debate over ‘know”s contextual variability, I claim, has been unnecessarily impoverished by the strong focus on simple sentences. Here, I wish to advocate going beyond the straightforward, beyond bank cases, airport cases, and their kin. In particular, I want to show that a wider look at the embedding behaviour of ‘know that’ in various linguistic environments can teach us a whole lot more about the mechanisms of context-sensitivity involved with ‘know that’. I think that semantic approaches to the context-sensitivity of ‘know-that’ have a much better chance of accounting for *embedded* knowledge sentences—occurrences of ‘*S* knows/doesn’t know that *p*’ are embedded in larger linguistic environments such as attitude reports, conditional sentences, comparatives, expressions of probability, etc.—than is often assumed. But I won’t argue for this here.

In this paper, I will take issue with a popular brand of invariantism, which accounts for context-sensitivity by adopting a pragmatic story. I will argue that it cannot give a plausible account of embedded knowledge sentences.

I start by sketching the point of departure from simple knowledge sentences and the most popular pragmatic account of ‘know”s contextual variability given by invariantists (Sect. 2). I’ll then introduce a paradigmatic example of an embedded occurrence of a knowledge sentence and show that it poses a threefold problem for pragmatic invariantism: the problem of embedded implicatures (Sects. 3, 4). Section 5 shows that the problem generalizes: the troubling data from embedded knowledge sentences is widespread and systematic. Next, I will sketch some challenges that any invariantist pragmatic strategy faces, suggesting that the prospects for pragmatic invariantism of any sort are dim (Sect. 6). I’ll close by reviewing the ramifications of my case against pragmatic invariantism for the methodology in our investigation of the meaning of ‘know that’ (Sect. 7).

One caveat before we start. I’m only concerned with the meaning of ‘know that’ here: occurrences of ‘know’ that are followed by a ‘that’-clause (or where a ‘that’-clause is deleted by ellipsis, or where ‘know’ is followed by an expression of propositional anaphora such as ‘it’, ‘this’, ‘that’).^1^ I do not make more general claims about the meaning of ‘know-wh.’

## Simple occurrences and pragmatic invariantism

### Simple variability

Let’s call clauses of roughly the form ‘*S* knows that *p*’ and ‘*S* doesn’t know that *p*’ *knowledge sentences*. A knowledge sentence can occur *simply*, i.e. unembedded, as in (1):
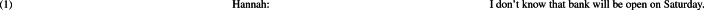


Knowledge sentences can also occur *embedded* in linguistic environments, e.g. under the attitude verb ‘believe’ as in (2):
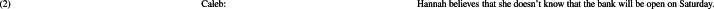


It is worth noting that knowledge sentences can occur in any construction that takes ‘that’-clauses as complements. We will come back to the systematic embeddability of knowledge sentences below.

Knowledge sentences, it is widely agreed, are contextually variable: the same sentence type can be used to communicate different things in different contexts. The dominant simple methodology tests for contextual variation by probing intuitive judgments of truth and falsity (felicity and infelicity^2^) of assertions of simple knowledge sentences, together with minimal-contrast vignettes that establish contextual settings. DeRose’s bank cases provide the paradigm. For our purposes, it’s well worth rehearsing them (here in Stanley’s presentation):

*Low Stakes.* Hannah and her wife Sarah are driving home on a Friday afternoon. They plan to stop at the bank on the way home to deposit their paychecks. It is not important that they do so, as they have no impending bills. But as they drive past the bank, they notice that the lines inside are very long, as they often are on Friday afternoons. Realizing that it isn’t very important that their paychecks are deposited right away, Hannah says, “I know the bank will be open tomorrow, since I was there just two weeks ago on Saturday morning. So we can deposit our paychecks tomorrow morning.”

*High Stakes.* Hannah and her wife Sarah are driving home on a Friday afternoon. They plan to stop at the bank on the way home to deposit their paychecks. Since they have an impending bill coming due, and very little in their account, it is very important that they deposit their paychecks by Saturday. Hannah notes that she was at the bank two weeks before on a Saturday morning, and it was open. But, as Sarah points out, banks do change their hours. Hannah says, “I guess you’re right. I don’t know that the bank will be open tomorrow.” (Stanley 2005, pp. 3–4)

It is widely judged that both Hannah’s knowledge attribution in *Low Stakes*, repeated in (3) below, and her knowledge denial in *High Stakes*, repeated in (4) below, are true (or felicitous),^3^ despite the fact that *Low Stakes* and *High Stakes* differ only in Hannah’s and Sarah’s practical stakes and Sarah’s raising of the possibility that banks change their hours.^4^
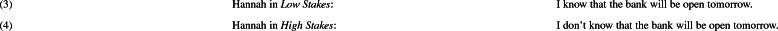


In addition, many accept that Hannah’s knowledge attribution in (5) would be false if made in *High Stakes*:
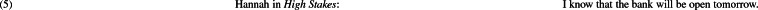


Let’s call this pattern of truth/falsity (felicity/infelicity) judgments *simple variability*.

### Moderate pragmatic invariantism (MPI) and simple variability

Simple variability has motivated many classical invariantists to complement their invariantism with a pragmatic account. This isn’t the only invariantist option to respond to simple variability. Other invariantists have adopted psychological error-theories,^5^ ‘warranted assertability maneuvers’,^6^ or subject-sensitive invariantism.^7^ These views are not my target in this paper, though I do think embedding data pose problems to many of them. Here, I will focus on classical invariantism and the pragmatic strategy of accounting for simple variability in terms of a (Gricean) implicature account. For concreteness, I’ll spin my discussion around Moderate Pragmatic Invariantism, and insofar as details matter, I’ll take issue with the account defended by Rysiew (2001, 2005, 2007) and Brown (2005, 2006). My arguments should apply, mutatis mutandis, to all versions of moderate pragmatic invariantism that take their cue from Gricean conversational implicatures.^8^ (This doesn’t mean the details of the views don’t matter, just that there are sufficiently adapted arguments like those in this paper against each of particular Gricean versions of MPI.)

Moderate pragmatic invariantism (MPI) is the combination of three theses:

(I) Invariantism: ‘Know-that’ semantically expresses an invariant meaning; its contribution to the content expressed by clauses in which it occurs does not vary with features of the context such as interests, purposes, stakes or salient error possibilities. Nor does the truth value of a clause ‘*S* knows [doesn’t know] that *p*’ at a context of use depend on such contextual features.
(M) Moderatism: The demands on a subject’s epistemic position to count as knowing are moderately low. Many ordinary simple uses of knowledge sentences are literally true.
(P) Pragmatic Content: Uses of knowledge sentences in High contexts of use, such as *High Stakes*, pragmatically convey a content that may be distinct from the semantic content expressed in that context.

Moderate invariantism (MI) should be sufficiently familiar, and it’s easy to see why it, by itself, isn’t sufficient to give all truth and falsity judgments in the bank cases their due. To see why not, note that by (I), all uses of knowledge sentences express the same invariant meaning and that by (M), this invariant meaning expresses moderately weak standards for knowledge. For simplicity, let’s call the content contributed to the proposition semantically expressed by ‘know’ in any context *know* $_{{\textsc {Weak}}}$. Thus, Moderate Invariantism has it that the knowledge claims (3), (4) and (5) express the following propositions, marked by *italics*:
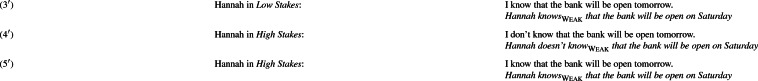


The proposition semantically expressed in $(3^{\prime })$ is true in *Low Stakes*, in line with speaker judgments. The trouble for Moderate Invariantism comes from the truth values of the propositions expressed by $(4^{\prime })$ and $(5^{\prime })$ in *High Stakes*. According to Moderate Invariantism, $(4^{\prime })$ is literally false and $(5^{\prime })$ is literally true, contrary to speakers’ judgments.

In order to square moderate invariantism with speaker judgments of truth regarding (4) and of falsity regarding (5), Moderate Pragmatic Invariantists hold that uses of knowledge sentences in *High Stakes* pragmatically convey a content distinct from the semantically expressed content. To a first approximation, this pragmatically conveyed content is about Hannah’s (not) being in an even stronger epistemic position than knowledge with regard to the bank’s being open on Saturday. According to Rysiew, Hannah’s claim (4), ‘I don’t know that the bank will be open tomorrow,’ in *High Stakes* conveys, inter alia, that Hannah cannot rule out the counter-possibilities to the bank’s being open on Saturday that are salient at the context of utterance, *High Stakes*. Since Sarah raised the possibility of banks changing their hours, the pragmatically conveyed content thus entails that Hannah cannot rule out the possibility that the bank in question has changed its hours since she has last been there on a Saturday. This content, and the entailment, are true in *High Stakes*. Similarly, the pragmatically conveyed content of Hannah’s knowledge attribution in (5) is that Hannah can rule out the counter-possibilities to the bank’s being open on Saturday that are salient at *High Stakes*, and this content is false in *High Stakes*. Hence, according to MPI it is pragmatic contents that are the targets of speakers’ judgments of truth and falsity, rather than (merely) the semantically expressed *know* $_{{\textsc {Weak}}}$-proposition.^9^

Before we move on to embedded uses, it is worth highlighting three relevant features of MPI. First, the pragmatic account relies on a Gricean picture of pragmatics. In particular, speakers can be assumed to be cooperative and obey the conversational maxims unless there is evidence to the contrary. This involves that speakers obey the maxim of Relation, which enjoins speakers to ‘be relevant’—that is, to make their conversational contribution relevant to the purpose and direction of the conversation at the stage at which it occurs (cf. Grice 1975, p. 27).

A second feature is that it is Relation that plays a central role in prompting the conveyance of a pragmatic content by Hannah’s knowledge claims in *High Stakes*. After Sarah has raised the possibility of bank’s changing their hours, Hannah may reasonably be expected to make her knowledge claims relevant to the question of whether or not this possibility obtains. But her knowledge claims taken literally do not address this question. According to Rysiew, who uses the relevant alternatives framework to spell out his view, *know* $_{{\textsc {Weak}}}$ implies being in a position to rule out the relevant alternatives. The *relevant* alternatives to any allegedly known proposition do not vary with non-traditional factors (such as practical stakes, interests, and salience of error possibilities) in context.^10^ Since in *High Stakes*, banks changing their hours is a salient but *not* a relevant alternative to the bank’s being open on Saturday, the propositions semantically expressed by Hannah’s knowledge claims don’t speak to the pertinent question. As Hannah can be assumed to be cooperative and obey Relation, however, hearers may infer that she intended to convey another content that does address the question—roughly that Hannah is / is not in a position to rule out the salient counter-possibilities to the bank’s being open on Saturday.

The third feature of the account is the status of the pragmatically conveyed content as a Gricean conversational implicature. Among the properties of conversational implicatures is calculability: hearers must be able to infer the implicated content from the speaker’s act of (literally) saying that *p*, available knowledge of the context as well as background knowledge, and the assumption of the speaker’s observation of the conversational maxims.

In sum, the pragmatic part of MPI is needed to account for speakers’ judgments of truth and falsity regarding knowledge claims made in contexts such as *High Stakes*. Let’s follow the oversimplifying tradition and call contexts in which epistemic standards are high (due to sceptical worries, salience of far-away counter-possibilities, or practical concerns) High. MPI’s pragmatic story for simple variability is Gricean: speakers in High semantically express one thing and pragmatically implicate another, which hearers could calculate by drawing on the speakers’ observation of the maxim of Relation, among other things.

## Embedded occurrences and the problem of embedded implicatures

Does the invariantist-cum-pragmatic story extend to all uses of knowledge sentences in assertions? Once we have surveyed a range of embedding constructions (Sect. 5), we will see that it fails dramatically. For now, let’s take small steps to see what the problem is. Consider an embedding of a knowledge sentence under the attitude verb ‘believe’. Suppose Caleb overhears Hannah’s knowledge denial (4) in *High Stakes*. Since he has no reason to assume Hannah is insincere, it seems that he can truly assert (2).
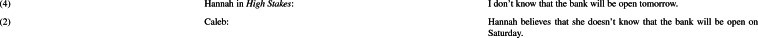


Can MPI account for the judgment of truth regarding Caleb’s report? According to MPI, (2) semantically expresses the content *that Hannah believes that she doesn’t know* $_{{\textsc {Weak}}}$ *that the bank will be open on Saturday*. Is this what (2) communicates? It seems not. First, this semantic content is likely false, contrary to the intuitive judgment of (2). As the bank cases are presented, Hannah hasn’t changed her mind between *Low Stakes* and *High Stakes*. [DeRose’s (2009, p. 2) presentation includes the words ‘Remaining as confident as I was before that the bank will be open then...’] And invariantists and their opponents have reason to say about *Low Stakes* that Hannah believes her knowledge$_{{\textsc {Weak}}}$ attribution. So it’s unlikely that she believes the negation in *High Stakes*. Second, it would be at odds with the Gricean picture to hold that Caleb is reporting a belief of Hannah’s with the content *that Hannah doesn’t know* $_{{Weak}}$ *that the bank will be open on Saturday* on the basis of overhearing (4). After all, according to MPI, Hannah’s assertion in (4) doesn’t communicate this false semantic content but instead the true, pragmatically implicated content *that Hannah cannot rule out the counter-possibilities salient in High Stakes*.^11^ As a general rule, sincere speakers intend to communicate what they believe. So if Hannah in (4) intends to communicate the pragmatically implicated content rather than the semantically expressed content, she expresses her belief in the pragmatically implicated content. (Compare: If, at the end of a terrible day, you make the ironical assertion ‘It doesn’t get any better than this’, you presumably don’t express a belief in its literal content. On the contrary, you probably disbelieve the literal content of your assertion and intend to express your belief in the opposite.^12^) So Caleb’s assertion is most naturally understood as reporting the belief Hannah intended to communicate, and this is the content (4) pragmatically implicates, not the content it semantically expresses.^13^

If (2) cannot be understood as communicating MPI’s semantic content, then the question becomes whether MPI can predict that Caleb’s belief report in (2) communicates the true content *that Hannah believes that she cannot rule out the counter-possibilities salient in High Stakes*. If so, then given MPI, (2) would have to be a case of embedded implicature: the alleged conversational implicature, which would normally be associated with an assertion of a particular clause, arises when that clause is uttered as an embedded constituent in a complex sentence (cf. Simons 2010). That is, in (2) the alleged implicature *that Hannah cannot rule out the counter-possibilities salient in High Stakes* is needed to arise even when the knowledge sentence falls under the scope of the attitude verb ‘believe’; it seems to arise *locally*—within the scope of ‘believe’.

There are three problems with an alleged embedded implicature for (2), which leave MPI without a plausible general story for embedded occurrences of knowledge sentences: the calculation problem, the Compositionality Problem, and the Context Problem. Together, they present a pernicious version of the problem of embedded implicatures. Note that the calculation problem and the Compositionality Problem arise for embedded conversational implicatures in general.^14^ So it’s not a surprise they would arise for a Gricean implicature account of knowledge claims. Still, it is informative to understand the details of the problems as they arise with embedded knowledge sentences. We will come back to the more general nature of these two problems in Sect. 6.

### The calculation problem

Since Caleb’s belief report in (2) isn’t plausibly interpreted as conveying its literal (semantic) invariantist content, MPI is forced to give a pragmatic-implicature account. (2) must be taken to report the belief Hannah expressed with her utterance in (4) (italics indicate that $(2^{\prime })$ represents a content):
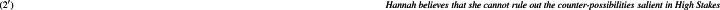


The first problem for MPI is that the content given by $(2^{\prime })$ cannot be inferred by speaker and hearers. The Problem of Calculation arises from the Gricean assumption of calculability.

- *Calculability*: Conversational implicatures must be calculable on the basis of (i) the speaker’s *act of saying that p* (semantic content), (ii) knowledge of the context as well as background knowledge, and (iii) the assumption of the speaker’s cooperativity (observation of conversational maxims).^15^

Grice’s notion of ‘saying’ is closely tied to conventional, semantic meaning (Grice 1975, p. 25); the act of saying can be understood, roughly, as the act of asserting literal content.^16^ Importantly, only full propositional content can be ‘said,’ or asserted. Proper parts of propositional content need not themselves be ‘said,’ or asserted. Asserting ‘If *q*, then *r*’ doesn’t involve asserting *q* or asserting *r*; asserting ‘*q* or *r*’ doesn’t involve asserting *q* or asserting *r*; asserting ‘A believes that *q*’ doesn’t involve asserting *q*. By Calculability, a conversational implicature can only be calculated from something that is said. It’s helpful to keep in mind that on the Gricean picture ‘saying what you say comes first; conversational implicatures come after’ (Simons 2010, p. 140). The Problem of Calculation is that only the semantic propositional content as a whole is said, and no conversational implicature can be calculated from any embedded clause whose semantic content is not itself said.^17^

In uttering ‘Hannah believes that she doesn’t know that the bank will be open on Saturday’ in (2), Caleb does not say (in the Gricean sense) that Hannah doesn’t know that the bank will be open. So no implicature can be calculated locally from the embedded clause ‘she doesn’t know that the bank will be open on Saturday’. This is the Problem of Calculation for MPI.

But if the implicature necessary for (2) cannot be calculated locally, perhaps it can be calculated globally from the entire asserted (semantic) content of the report? The challenge for any attempt to account for the implicature globally is to predict just the right implicature. I will postpone a full discussion to Sect. 4. There we will see that global accounts suffer from the Problem of Context and other serious problems. Anticipating the failure of global implicatures for now, let’s move on to another problem with embedded implicatures for ‘know that.’^18^

### The compositionality problem

The compositionality problem arises given two orthodox assumptions:

- *Compositionality*: The meaning of a complex expression is determined by its structure and the meanings of its constituents.
- *Semantics/Pragmatics Interface*: Processes of composition happen at the level of *semantic* meaning and are independent of processes of pragmatic inferencing. In Gricean terms: what is said is determined compositionally from conventional meanings and is independent from what is conversationally implicated.

The problem with alleged embedded implicatures is that they would require an embedding expression to compose with the pragmatically inferred content of the embedded clause, so that pragmatic content would contribute to the output of the compositional process. This violates the assumption about the semantics/pragmatics interface.

As concerns (2), the meaning of ‘Hannah believes’ would have to compose with the pragmatically implicated content of ‘that she doesn’t know that the bank will be open on Saturday’. By the two above assumptions, however, the semantic meaning of the embedding expression ‘Hannah believes’ can only compose with the *semantic* meaning of the embedded clause to yield the literal, semantic content of (2), which is different from the communicated $(2^{\prime })$. The Problem of Compositionality for MPI is the problem that attitude verbs and other embedding expressions can only compose with the literal, invariant meaning of an embedded occurrences of ‘know that’.

It is worth highlighting that the Calculation and Compositionality Problems arise for *every* embedded occurrence of ‘know that’ which needs to receive a stronger-than-knowledge$_{{\textsc {Weak}}}$ reading.^19^ The next problem, the Context Problem, is specific to cross-contextual attitude- and speech act-reports.^20^

### The context problem

The context problem arises from a Gricean assumption about context involved in *Calculability*:

- *Context*: The calculation of implicatures requires, if any, factors available to speakers and hearers in the *context of use*.

A context of use is a possible occasion of use of a sentence; for a given speech act, its context of use is the situation in which the speech act using the sentence is made.^21^ It follows from *Context* that if speaker or hearers in a context of use lack some information required for the calculation of an alleged conversational implicature, then they cannot infer the implicature. Note, next, that MPI’s exploitation of the maxim of Relation for Hannah’s knowledge denial in (4) requires that it be an (implicit) concern in the context of use whether *Hannah* can rule out the salient counter-possibilities to the bank’s having changed its hours. The Problem of Context regarding (2) then is that the context of use of Caleb’s report in (2) doesn’t provide the factors that would yield the needed communicated content in $(2^{\prime })$. This can take two forms, depending on the assumptions we make about Caleb’s reporting context.

First, assume that no counter-possibilities to the bank’s being open on Saturday are of any concern in Caleb’s reporting context. (Suppose Caleb has only overheard Hannah’s assertion in *High Stakes* but hasn’t heard Sarah’s mention of banks changing their hours, nor does he know that it’s important to Hannah and Sarah to deposit their checks by Saturday.) Then no implicature is derived, since Caleb and his hearers are in no position to calculate an implicature (setting aside the Calculation and Compositionality Problems) whose calculation requires the salience of any counter-possibilities to the bank’s being open that go beyond the relevant alternatives. The only concern in Caleb’s context might be what Hannah believes. Still, it seems that Caleb can truly make the report in (2) on the basis of Hannah’s knowledge denial in *High Stakes*.

Second, assume that in Caleb’s conversation, the possibility that the entire bank has gone bankrupt and closed all branches permanently is salient. Then, *if* there was an implicature from (2), it would have to be this: *Hannah believes that she cannot rule out the counter-possibility that the bank has gone bankrupt and closed all branches permanently*. Put more crudely, the implicature would be something like *that Hannah believes that she cannot rule out the counter-possibilities that are salient to the reporter*. But Hannah presumably doesn’t have a belief about being able to rule out counter-possibilities salient at some reporting context. On these assumptions about the reporting context, MPI would predict the wrong implicature.^22^

The Context Problem reveals that unless the salient-to-Hannah counter-possibilities are also salient in Caleb’s reporting context, MPI’s Gricean account cannot deliver the needed content of $(2^{\prime })$.

A note before we move on: the Context Problem arises for uses of knowledge sentences in cross-contextual attitude- and speech-reports as well as in embeddings under reportative evidentials such as ‘reportedly’, ‘supposedly’, and ‘apparently.’ It has been pointed out that epistemic contextualism faces a problem with such cross-contextual reports,^23^ so it seems that the problem can’t be due to the particularities of MPI. In fact, it is not surprising MPI would have the Context Problem, given that its early proponents Brown and Rysiew were looking for views that would deliver the contextualist’s predictions. But while indirect speech- and attitude reports may present a more general problem to several views, the resources those views can draw on to solve their problem depend on the assumptions and explanatory mechanisms specific to the views. For contextualists, the challenge is to explain how a context-sensitive expression like ‘know that’ can have their variable value bound by or shifted to the reported context of speech (or the context of the attitude holder in attitude reports) when the expression occurs within the scope of a verb like ‘say’ or ‘believe.’ And semantic proposals to meet this challenge on behalf of contextualism have been made (see, e.g. Huvenes 2012, ch. 5). Whether MPI can meet the challenge by appeal to Gricean mechanisms is an open question. I have tried to show that the likely answer is no.^24^

## Global Gricean implicatures?

Before we move on to see that the problem of embedded implicatures for MPI isn’t limited to belief reports, let’s pause to consider the response that MPI’s needed implicatures can be calculated globally. A way to save MPI in its Gricean form might come from avoiding the need for locally calculated, embedded implicatures that would have to enter into the compositional process. The Problem of Calculation, we noted, is only a problem for MPI if there is no way for speaker and hearers to calculate the alleged implicature globally—that is, from the act of saying the semantic content of the whole sentence, including the semantic contribution of embedding and embedded expressions. *If* there is a plausible account of how speaker and hearers come to calculate the alleged implicature globally in all cases where MPI needs it, then the Problem of Calculation and the Problem of Compositionality (which MPI faces only if the implicature is to arise locally) are avoided.

With regard to (2), the challenge for a global-calculation strategy for MPI is to explain how speaker and hearers come to calculate $(2^{\prime })$ rather than some other content.
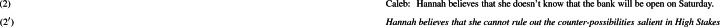


The global-implicature strategy faces three problems. The first problem is the Problem of Context once more. MPI’s pragmatic account is based on the maxim of Relation and the salience of counter-possibilities in the context of use. So unless the counter-possibility of banks changing their hours is not only salient in Hannah’s *High Stakes* but also salient in Caleb’s reporting context, speaker and hearers lack a crucial piece of information that would trigger the calculation of any implicature, or possess a salient piece of information that leads them to calculate the wrong implicature. The Context Problem is independent from the local or global nature of the calculation.

The second problem is that, even if we bracket the Context Problem by supposing that the pertinent counter-possibility is salient in Caleb’s reporting context, MPI’s Relation-based account would get an implicature at the wrong ‘level.’ If Caleb was himself in a High context where it was relevant (in the sense of the maxim ‘Be relevant’) whether or not the bank had changed its hours, the implicature from (2) that most directly addresses this concern would be *that Hannah cannot rule out that the bank has changed its hours* (rather than that Hannah believes this). This implicature is more relevant to the salient issue than the local implicature that Hannah believes that she cannot rule out that the bank has changed its hours. The second problem thus is that we would need a convincing account not only of why $(2^{\prime })$ is implicated but also of why the content *that Hannah cannot rule out that the bank has changed its hours* is not implicated. What’s worse, this account would have to be principled enough to also deliver the right local implicatures for knowledge sentences in all embedding constructions.

The third problem is as follows. Perhaps moderate pragmatic invariantist should give a different account of the global calculation of $(2^{\prime })$. Perhaps they can appeal to the Gricean maxim of Quality—‘Try to make your contribution one that is true’ (Grice 1975). Speaker and hearers might infer the implicature from the fact that Caleb makes an assertion whose literal, semantic content is false and from the assumption that Caleb is still trying to be cooperative and, by observing Quality, contribute something true. By further steps of reasoning, hearers might then arrive at the conclusion that Caleb intended to communicate the stronger, true content $(2^{\prime })$.

The problem with this strategy is that (i) we would still need a story of why hearers come to infer the right implicature from the above assumptions, a story that doesn’t inherit the above problems for Relation + salience; and that (ii) the strategy doesn’t suitably generalize. Suppose Hannah in *High Stakes* had made the knowledge attribution in (5), repeated below. Then it seems Caleb could report (6):
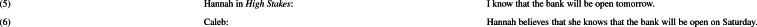


The semantic content expressed by Caleb’s report, according to MPI, is the proposition given by (6’).
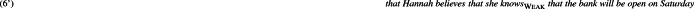


So (6) is literally true on MPI, because Hannah does believe that she knows$_{{\textsc {Weak}}}$ if she believes in *High Stakes* that she can rule out the counter-possibilities salient to her (which is what she communicates with her speech act in (5)). So Quality is observed and its apparent violation cannot trigger the calculation of an implicature. But neither can (6) be a case of Relation-triggered implicature: the two above problems arise here, too. And while (6’) is true of Hannah, it doesn’t report Hannah as having the belief she expresses in asserting (5) in *High Stakes*.

In sum, the global-implicature strategy isn’t promising. The three problems rule out a global-implicature strategy as a viable option for predicting the needed readings for all instances of embedded knowledge sentences.

## Generalizing the problem

The calculation, compositionality, and context problems—in short, the problem of embedded implicatures—for knowledge sentences embedded under attitude verbs raise serious doubt about the viability of defending invariantism by appeal to Gricean pragmatics. But the problem isn’t limited to embeddings under attitude verbs (though the context problem arises only in some embedding environments). ‘Know that’ embeds naturally in all kinds of constructions, and for each we can find uses that require a reading stronger than the invariant *know* $_{{\textsc {Weak}}}$ as well as uses that do not require a stronger reading. Consider the following occurrences in conditional sentences, comparatives, ‘only’ and expressions of probability, to pick just a few familiar ones for illustration.

Conditional sentences—‘know’ in the antecedent:
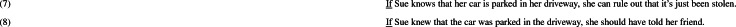


Conditional sentences—‘know’ in the consequent:
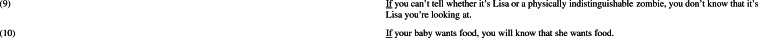


Comparatives:
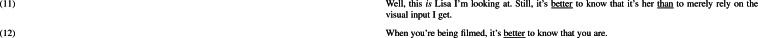


‘Only’:
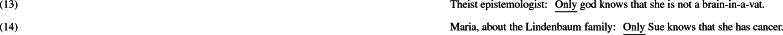


Probability talk:
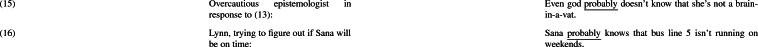


Observe, first, that ‘know that’ embeds flawlessly in all these constructions; (7)–(16) all have a natural felicitous reading.

Observe, second, that the second example of each of the pairs (7)/(8) through (15)/(16) has a true natural reading that we can call a *weak reading*: there is a true reading of the sentence on which ‘know’ can be understood as contributing the moderate invariantist’s semantic content *know* $_{{\textsc {Weak}}}$. For instance, (8) ‘If you knew that the car was parked outside, you should have told me’ can be heard as felicitous even when ‘know’ imposes meetably low demands on the addressee’s past epistemic position.

In contrast, observe third that the first example of each pair requires a reading of ‘know’ stronger than *know* $_{{\textsc {Weak}}}$. For illustration, consider the conditional sentence in (7), ‘If Sue knows that her car is parked in the driveway, she can rule out that it’s just been stolen’, an example wrought from familiar material.^25^ If an assertion of (7) expressed the (moderate invariantist’s semantic) content *that if Sue knows* $_{{\textsc {Weak}}}$ *that her car is parked in the driveway, she can rule out that it’s just been stolen*, it should ring false: moderate knowledge$_{{\textsc {Weak}}}$ does not put one in a position to rule out that the car one parked in the driveway a while ago has been stolen in the meantime (or so it is assumed by moderatism). In order to get the natural true reading *that if Sue knows* $_{{\textsc {Strong}}}$ *that her car is parked in the driveway, she can rule out that it’s just been stolen* (where ‘knows$_{{\textsc {Strong}}}$’ is our dummy stand-in for the stronger notion of knowledge that according to MPI is generated by implicature), MPI needs to claim that the implicature is calculated locally within the ‘if...’-clause and that it composes with the literal meaning of the consequent of the conditional sentence. But this raises the Problems of Calculation and Compositionality (as well as problems with a global-implicature strategy) over again. The same holds for all of the second examples of the above pairs.

It isn’t hard to find examples of embedded knowledge sentences in all kinds of constructions whose intuitively true assertion requires a *strong reading* of ‘know’—an interpretation of ‘know’ that is stronger than the moderate content that, according to MPI, ‘know’ semantically contributes. So the moral is that the problem of embedded implicatures for MPI generalizes: On a whole variety of constructions embedding knowledge sentences, some uses in context would require, given MPI, that the knowledge sentences contribute a local implicature to the overall communicated content.

## Further pragmatic options

If correct, the problem of embedded implicatures sinks extant versions of Moderate Pragmatic Invariantism, which appeal to a Gricean picture of pragmatics. So what are the options for MPIers?

First, proponents of MPI may point out that at least the Calculation Problem and Compositionality Problems beset the traditional Gricean account of conversational implicatures in general—no matter the relevant expressions used in embeddings; so the Context Problem aside, the problem isn’t specific to ‘know.’ But while this observation is correct, it is not clear that it is good news for extant versions of MPI. One lesson to draw from the observation would be a wholesale abandonment of all versions of MPI that rely on the traditional Gricean account of conversational implicatures. But as a matter of fact, extant versions of MPI for the most part do rely on Gricean pragmatics; there aren’t any worked out moderate invariantist pragmatic alternatives.^26^

Another lesson to draw from the observation, would be to look for non-Gricean alternatives. This is the second option for MPIers. There is a wide range of non-Gricean pragmatic accounts that have been developed in reaction to the Gricean problems with embedded implicatures. Most of these have been developed for particular phenomena, such as scalar implicatures. Non-Gricean pragmatic accounts include views that posit non-Gricean implicatures—e.g. Levinson’s ‘default implicatures’ and Chierchia’s and Landman’s implicatures that are part of grammar—as well as views that posit prior, ‘primary’ pragmatic processes—e.g. relevance theory’s ‘explicatures’ (Sperber and Wilson), Bach’s ‘implic*i*tures,’ and Recanati’s ‘free enrichment’ (Levinson 2000; Chierchia 2004; Landman 2000; Sperber and Wilson 1986; Recanati 2010). So we may ask: Can there be a non-Gricean version of MPI on the basis of any such pragmatic account that successfully explains the contextual variability and embeddability of ‘know’?

This isn’t the place to preemptively argue in a systematic fashion against every single possible non-Gricean option for MPIers. I will leave it as a challenge to proponents of MPI to develop the details of a plausible non-Gricean version of MPI. But I’d like to offer three main reasons why any attempt at combining Moderate Invariantism with a non-Gricean pragmatic account is unlikely to succeed.

First, the above-mentioned pragmatic accounts are likely to involve explanatory features that just don’t fit the behaviour of ‘know’. For instance, non-Gricean accounts developed for scalar implicatures are accounts of additive implicatures: speakers convey an implicature *in addition to* semantic content; an assertion of ‘Some students did well’ conveys the semantic content that at least one student (and possibly all) did well, *in addition* the assertion regularly conveys the content that not all students did well. But an account tailor-made for additive implicatures won’t do for MPI. For instance, MPI needs to posit a substitutional implicature to fully account for the simple variability of (4), ‘I don’t know that the bank will be open tomorrow.’ With a knowledge denial in High, a speaker conveys the implicature *instead of* semantic content.^27^ So the challenge is to show that these accounts do in fact apply to the case of ‘know.’

Secondly, it needs to be shown that adopting a particular non-Gricean pragmatic account does indeed solve the problem of embedded implicatures for the case of ‘know.’ While extant non-Gricean accounts have been developed largely in response to Calculation and Compositionality Problems, all of these accounts are likely to accept the assumption of *Context* (see Sect. 3.3 above). So they do not solve MPI’s Context Problem.

Thirdly, the main post-Gricean pragmatic accounts of embedded implicatures aren’t a good fit with the overall *invariantist* view of the meaning of ‘know.’ On one group of pragmatic views, such as Recanati’s (2004, 2010) ‘truth-conditional pragmatics’ and Sperber and Wilson’s (1986) relevance theory, ‘know’ would be likened to expressions that are standardly conceived to have contextually variable meanings, leading to a ‘radical contextualist’ account of ‘know.’ In this vein, Stainton (2010) and Pynn (2015) appeal to Recanati’s notion of ‘free enrichment’ in defence of a pragmatic version of epistemic contextualism, not in defence of invariantism.^28^ On another group of non-Gricean views, such as Chierchia’s (2004) and Landman’s (2000) grammatical views, ‘know’ would generate default implicatures as part of the semantic composition process in grammar, leaving no distinguished, implicature-free level of invariant semantic meaning for ‘know.’ Finally, on Levinson’s (2000) account of ‘default implicatures’, ‘know”s implicatures would be *conventionalized*: like other default implicatures, they would be associated with a certain linguistic item, ‘know’, that serves as a trigger for the automatic process of implicature generation; this process leads to an intermediate layer of meaning between sentence meaning and speaker meaning. Here one might wonder what makes the layer of sentence meaning, at which ‘know’ could be said to have a single, invariant meaning, the primary target of conceptual analysis into ‘know”s meaning, if implicatures are generated by default and are conventionalized. So neither of the main post-Gricean alternatives cleanly delivers the kind of invariant view on which ‘know’ has a single, privileged semantic meaning—*knowledge*. Instead, ‘know’ would have to be seen as being capable of expressing two or more meanings, neither of which is *the* privileged meaning capturing what is important about knowledge.

These challenges, I contend, leave dim prospects for rescuing moderate invariantism with a pragmatic story of ‘know”s contextual variability in both embedded and unembedded uses.

## Conclusion

Moderate pragmatic invariantism was developed as a response to the contextualist challenge from the contextual variability of ‘know.’ In order to account for the variation of speakers’ judgments of truth and falsity, or felicity and infelicity, of simple knowledge sentences from one context to the next, moderate invariantism was supplemented with a pragmatic account. Here, I have left open whether the Gricean pragmatic account favoured by moderate invariantists is successful in explaining the contextual variation found with *simple* occurrences of knowledge sentences.^29^ At any rate, this wouldn’t be sufficient. By shifting attention to embedded occurrences of ‘know that’—e.g. under attitude verbs, in conditional sentences, comparatives, ‘only’, expressions of probability, asf.—I have argued that MPI faces a particularly pernicious form of the problem of embedded implicatures. The linguistically flawless embedding of knowledge sentences in a whole variety of standard embedding environments underlines the paper’s negative conclusion: Gricean versions of MPI cannot give a plausible account of the whole variety of embedded knowledge sentences, and the prospects for non-Gricean developments of MPI aren’t bright.

The arguments in this paper have targeted moderate pragmatic invariantism, in particular the versions defended by Brown (2005, 2006) and Rysiew (2001, 2005, 2007). It should be understood, however, that they apply, mutatis mutandis, to other versions of pragmatic invariantism, including sceptical pragmatic invariantism,^30^ provided that these views endorse assumptions about the inference processes for implicatures, the semantics/pragmatics interface, and context similar to the ones that gave rise to the calculation, compositionality, and context problems for MPI. But even versions of sceptical pragmatic invariantism that deny these assumptions would still need to answer the challenges outlined in Sect. 6.

Of course, invariantists may wish to account for contextual variation of any sort, whether with embedded or simple occurrences of knowledge sentences, by endorsing a psychological error theory.^31^ Nothing I have said here rules out this option. Given the arguments against pragmatic invariantism presented here, this may in fact be the invariantist’s best bet.

Most of the debate over the contextual variation of ‘know that’ has focused on simple occurrences of knowledge sentences. While there is nothing amiss with a ‘methodology of the straightforward’ that works from clear judgments on easy sentences involving the expression under investigation, the arguments in this paper suggest that venturing beyond simple occurrences and looking at the embedding behaviour of ‘know that’ can teach us a whole lot more about the context-sensitivity of ‘know that.’

## Footnotes

1 An example: A says ‘Lucy works for GM’, B replies ‘I know’ or ‘I know that’.

2 It won’t matter much to us here whether there is an empirically robust distinction between speakers’ judgments of (semantic, literal) truth and falsity and their judgments of (pragmatic) felicity and infelicity. I assume that there isn’t, so it’s the theoretician’s task to argue for a semantic over a pragmatic account, or for a pragmatic over a semantic account, that does justice to speaker judgments. For brevity, I will talk of speakers’ judgments of truth and falsity below, but this should be understood in a loose sense as encompassing speakers’ answers to questions such as ‘Do you agree or disagree with Hannah’s claim?’ I do not intend my use to prejudge whether speaker judgments track (semantic/literal) truth/falsity rather than felicity/infelicity of the speech act in context.

3 More terminology: I use ‘knowledge attribution’ for an assertive use of a unnegated knowledge sentence and ‘knowledge denial’ for an assertive use of a negated knowledge sentence. ‘Knowledge claim’ is the term that covers both knowledge attributions and denials in my sense. The three labels thus refer to speech acts.

4 Experimental evidence of folk judgments along these lines is found by Schaffer and Knobe (2012) using cases adapted from DeRose’s bank case. Contextual variation in judgments of simple knowledge claims is not found as clearly in studies by Buckwalter (2010) and May et al. (2010), though. See Buckwalter (2017) and Gerken (2017, Ch. 2) for an overview of experimental work. In what follows, I’m going to assume that speaker judgments in paradigm pairs of cases such as the bank cases provide *sufficient* evidence for contextual variation.

5
Gerken (2012b, 2013), Nagel (2010a, b), and Williamson (2005).

6
Brown (2010), Gerken (2012a), Goldberg (2015), Greenough (2011), Levin (2008), McKinnon (2013), Rescorla (2009), and Stone (2007).

7
Fantl and McGrath (2002, 2009), Hawthorne (2004), Stanley (2005), and Weatherson (2005).

8 These versions of MPI also include Black (2005), Hazlett (2009), Pritchard (2010), and Locke’s (2017) and Lutz’s (2014) pragmatic-encroachment-based versions of MPI.

9 With regard to sentence meaning, I use ‘proposition’ and ‘content’ interchangeably throughout. I also sometimes use ‘content’ to refer to sub-sentential meaning, as in ‘the content semantically contributed by ‘know’.’

10 The ‘relevance’ of relevant alternatives theory invoked by Rysiew has nothing to do with the ‘relevance’ required by Grice’s maxim of Relation.

11 The pragmatic implicature of (4) is substitutional; that is, the pragmatic implicature is communicated *instead of* (rather than in addition to) the semantically expressed content [see Meibauer (2009, p. 374) and Kindermann (2016, p. 437) for the distinction between substitutional vs additive implicatures]. If (4)’s semantic content was communicated, too, MPI couldn’t account for the judgment of truth: the semantic content, even when in conjunction with the pragmatic implicature, is false in *High Stakes*.

12 Cases of metaphor, hyperbole and irony are apt comparisons within a Gricean framework, since they also belong to the class of substitutional implicatures. See Sect.  6 and especially footnote 27 on substitutional vs additive implicatures.

13 Some invariantists might wish to pursue the literal-interpretation line nonetheless. This line faces two further obstacles. First, the invariantist needs to provide a plausible explanation of Hannah’s error, of why her reported belief is a false belief in *High Stakes* despite (her) appearance to the contrary. But once an error theory is needed, the question is why that error theory isn’t explanatory of simple uses in all *High Stakes* cases, making the pragmatic account redundant. Second, the literal interpretation yields incorrect truth-value predictions for a whole variety of embedding constructions, discussed in Sect. 5 below. Again, a drastic error theory would be needed.

14 The terms ‘calculation problem’ and ‘compositionality problem’ are from Simons’ (2010, 2012) lucid presentation of the problem of embedded implicatures for Gricean conversational implicatures. My presentation follows hers in crucial aspects. See also Chierchia (2004) on the problem arising with scalar implicatures.

15 Cf. Grice (1975, p. 31)

16 Cf. Simons (2010, pp. 140–141) and Neale (1992)

17 The problem was already pointed out for embedded Gricean implicatures more generally by Cohen (1971); see also Recanati (2003).

18 It is instructive to contrast the calculation problem with the problem of third-person knowledge attributions, put forward against Rysiew’s version of MPI by MacFarlane (2005). Both problems use occurrences of knowledge sentences in particular constructions to show that MPI’s account of the calculation of the implicature fails. (Thanks to an anonymous referee for bringing the analogy to my attention.) In short, MacFarlane’s objection says that Rysiew’s (2001) Gricean-implicature explanation of first-person knowledge attributions doesn’t carry over to third-person cases. In response to the problem, Rysiew (2007, pp. 637–638) argued that a third-person knowledge attribution of the form ‘*S* knows that *p*’ conveys that *p* by the factivity of ‘know;’ and once it is conveyed that *p*, an implicature that the speaker knows that *p* can be calculated, in the same way Rysiew explains first-person cases (cf. also Lutz 2014, p. 1729). In contrast to MacFarlane’s problem, note that the calculation problem is more fundamental. It’s not just the objection that MPI’s Gricean implicature-calculation does not apply to the cases presented, but the point that no local Gricean calculation is possible from an embedded knowledge sentence that isn’t itself ‘said’ in the Gricean sense. The difference between MacFarlane’s problem and the calculation problem can also be seen from the fact that Rysiew’s (2007) account fails, even if successful for third-person knowledge attributions, for many embedding constructions, such as (2): These do not convey that the speaker knows what the that-clause of the embedded knowledge sentence expresses. ‘Know”s factivity does not project out of ‘believe that’ constructions: ‘*S* believes that she knows that *p*’ presupposes (or otherwise conveys) that *S* believes that *p* but it doesn’t presuppose that *p*. So an assertion of ‘*S* believes that she knows that *p*’ does not convey that *p*, and no implicature as to the speaker’s knowledge is generated.

19 Cf. Recanati (2003) and Simons (2010, 2012) for presentations of these problems arising for a wide range of alleged embedded implicatures.

20 Some embedded occurrences of knowledge sentences under reportative evidentials such as ‘reportedly’, ‘supposedly’, and ‘apparently’ face the Context Problem, too.

21 Cf. Kaplan’s notion of ‘context’ (1989, p. 494).

22 This second aspect of the Context Problem depends on the assumption that the implicated content is specific with respect to salient counter-possibilities. Rysiew makes this assumption, but other proponents of MPI choose less specific implicatures, such as that *S* ‘is not in a very strong epistemic position with respect to the relevant proposition’ (Brown 2006, p. 426). Thanks to Alexander Dinges for pointing this out to me.

23 See for instance Hawthorne (2004), Cappelen and Lepore (2005), and Brogaard (2008).

24 Thanks to an anonymous referee for pointing out the more general nature of the problem and the ramifications for the dialectical situation between MPI and contextualism.

25 See for instance Hawthorne (2004, p. 4) and MacFarlane (2005, p. 200).

26 Note however that Brown (2005) and Rysiew (2001, 2007) have expressed sympathy with Bach’s notion of implic*i*ture. Even their accounts, however, do not present us with sufficient detail to witness a departure from Gricean pragmatics significant enough to see their accounts liberated from the problem of embedded implicatures. See Blome-Tillmann (2013, Sect. 7) for arguments that the invariantist’s alleged implicatures cannot be cases of Bachian implic*i*tures.

27 In the case of additive implicatures, a speaker conveys what she says (semantic content) as well as something else. In the case of substitutional implicatures, the speaker doesn’t convey what the sentence used says in that context but only conveys something else in its place. See, e.g. Dinges (2015, p. 56) , Kindermann (2016, p. 437f.), and Meibauer (2009, p. 374) for the distinction.

28 See also Ludlow (2008) for a pragmatic version of contextualism. On Sperber and Wilson’s (1986) view of ‘explicatures’, ‘know’ would be in need of ‘filling out’ in order to make the expressed proposition complete and hence truth-evaluable. So the lexically encoded, purely semantic meaning of ‘know’ would be an incomplete meaning—hardly what invariantists want to claim. Cf. Blome-Tillmann’s (2013) arguments against invariantist appeals to Bachian implicitures for a similar problem.

29 See Blome-Tillmann (2013), Dimmock and Huvenes (2014) and Kindermann (2016) for reasons to believe that pragmatic invariantism doesn’t provide a plausible account even for simple occurrences of knowledge sentences.

30 See, e.g. Davis (2007) and Schaffer (2004) for developments of sceptical pragmatic invariantism.

31 See, for instance, the appeal to an ‘availability’ bias by Hawthorne (2004) and Williamson (2005); Nagel’s (2010a, b) notions of ‘epistemic egocentrism’ and ‘epistemic anxiety’; and Gerken’s (2012b, 2013) development of ‘epistemic focal bias.’

## References

[CR1] Black T (2005). Classic invariantism, relevance and warranted assertability manœvres. The Philosophical Quarterly.

[CR2] Blome-Tillmann M (2013). Knowledge and implicatures. Synthese.

[CR3] Brogaard B (2008). In defence of a perspectival semantics for ‘know’. Australasian Journal of Philosophy.

[CR4] Brown J (2005). Adapt or die: The death of invariantism?. The Philosophical Quarterly.

[CR5] Brown J (2006). Contextualism and warranted assertibility manoeuvres. Philosophical Studies.

[CR6] Brown J (2010). Knowledge and assertion. Philosophy and Phenomenological Research.

[CR7] Buckwalter W (2010). Knowledge isn’t closed on saturday: A study in ordinary language. Review of Philosophy and Psychology.

[CR8] Buckwalter W, Ichikawa JJ (2017). Epistemic contextualism and linguistic behavior. The Routledge handbook of epistemic contextualism.

[CR9] Cappelen H, Lepore E (2005). Insensitive semantics.

[CR10] Chierchia G, Belletti A (2004). Scalar implicature, polarity phenomena, and the syntax/pragmatics interface. Structures and beyond: The cartography of syntactic structures.

[CR11] Cohen LJ, Bar-Hillel Y (1971). Some remarks on Grice’s views about the logical particles of natural language. Pragmatics of natural languages.

[CR12] Davis W (2007). Knowledge claims and context: Loose use. Philosophical Studies.

[CR13] DeRose K (2009). The case for contextualism: Knowledge, skepticism, and context.

[CR14] Dimmock P, Huvenes T (2014). Knowledge, conservatism, and pragmatics. Synthese.

[CR15] Dinges A (2015). Innocent implicatures. Journal of Pragmatics.

[CR16] Fantl J, McGrath M (2002). Evidence, pragmatics, and justification. The Philosophical Review.

[CR17] Fantl J, McGrath M (2009). Knowledge in an uncertain world.

[CR18] Gerken M (2012). Discursive justification and skepticism. Synthese.

[CR19] Gerken M, Brown J, Gerken M (2012). On the cognitive bases of knowledge ascriptions. Knowledge ascriptions.

[CR20] Gerken M (2013). Epistemic focal bias. Australasian Journal of Philosophy.

[CR21] Gerken M (2017). On folk epistemology. How we think and talk about knowledge.

[CR22] Goldberg SC (2015). Assertion. On the philosophical significance of assertoric speech.

[CR23] Greenough P, Brown J, Cappelen H (2011). Truth-relativism, norm-relativism, and assertion. Assertion.

[CR24] Grice P, Cole P, Morgan J (1975). Logic and conversation. Syntax and semantics 3: Speech acts.

[CR25] Grice P (1989). Studies in the way of words.

[CR26] Hawthorne J (2004). Knowledge and lotteries.

[CR27] Hazlett A (2009). Knowledge and conversation. Philosophy and Phenomenological Research.

[CR28] Huvenes, T. T. (2012). *On the contrary: Disagreement, context, and relative truth*. Ph.D. thesis, University of St Andrews and University of Oslo.

[CR29] Kaplan D, Almog J, Perry J, Wettstein H (1989). Demonstratives. An essay on the semantics, logic, metaphysics, and epistemology of demonstratives and other indexicals. Themes from Kaplan.

[CR30] Kindermann D (2016). Knowledge, pragmatics, and error. Grazer Philosophische Studien.

[CR31] Landman F (2000). Events and plurality.

[CR32] Levin J (2008). Assertion, practical reason, and pragmatic theories of knowledge. Philosophy and Phenomenological Research.

[CR33] Levinson SC (2000). Presumptive meanings: The theory of generalized conversational implicature.

[CR34] Locke D (2017). Implicature and non-local pragmatic encroachment. Synthese.

[CR35] Ludlow P (2008). Cheap contextualism. Philosophical Issues.

[CR36] Lutz M (2014). The pragmatics of pragmatic encroachment. Synthese.

[CR37] MacFarlane J, Gendler TS, Hawthorne J (2005). The assessment-sensitivity of knowledge attributions. Oxford studies in epistemology 1.

[CR38] May J, Sinnott-Armstrong W, Hull JG, Zimmerman A (2010). Practical interests, relevant alternatives, and knowledge attributions: An empirical study. Review of Philosophy and Psychology.

[CR39] McKinnon R (2013). The supportive reasons norm of assertion. American Philosophical Quarterly.

[CR40] Meibauer J, Mey JL (2009). Implicature. Concise encyclopedia of pragmatics.

[CR41] Nagel J (2010). Epistemic anxiety and adaptive invariantism. Philosophical Perspectives.

[CR42] Nagel J (2010). Knowledge ascriptions and the psychological consequences of thinking about error. The Philosophical Quarterly.

[CR43] Neale S (1992). Paul Grice and the philosophy of language. Linguistics and Philosophy.

[CR44] Pritchard D, Keim-Campbell J, O’Rourke M, Silverstein H (2010). Contextualism, skepticism, and warranted assertability maneuvres. Knowledge and skepticism.

[CR45] Pynn G (2015). Pragmatic contextualism. Metaphilosophy.

[CR46] Recanati F (2003). Embedded implicatures. Philosophical Perspectives.

[CR47] Recanati F (2004). Literal meaning.

[CR48] Recanati F (2010). Truth-conditional pragmatics.

[CR49] Rescorla M (2009). Assertion and its constitutive norms. Philosophy and Phenomenological Research.

[CR50] Rysiew P (2001). The context-sensitivity of knowledge attributions. Noûs.

[CR51] Rysiew P (2005). Contesting contextualism. Grazer Philosophische Studien.

[CR52] Rysiew P (2007). Speaking of knowing. Noûs.

[CR53] Schaffer J (2004). Skepticism, contextualism, and discrimination. Philosophy and Phenomenological Research.

[CR54] Schaffer J, Knobe J (2012). Contrastive knowledge surveyed. Noûs.

[CR55] Simons M, Petrus K (2010). A gricean view on intrusive implicatures. Meaning and analysis: New essays on Grice.

[CR56] Simons M, Maienborn C, von Heusinger K, Portner P (2012). Implicature, chapter 92. Semantics: An international handbook of natural language meaning.

[CR57] Sperber D, Wilson D (1986). Relevance: Communication and cognition.

[CR58] Stainton R, Campbell J, O’Rourke M, Silverstein H (2010). Contextualism in epistemology and the context-sensitivity of ’knows’. Knowledge and skepticism.

[CR59] Stanley J (2005). Knowledge and practical interests.

[CR60] Stone J (2007). Contextualism and warranted assertion. Pacific Philosophical Quarterly.

[CR61] Weatherson B (2005). Can we do without pragmatic encroachment?. Philosophical Perspectives.

[CR62] Williamson T (2005). Contextualism, subject-sensitive invariantism, and knowledge of knowledge. The Philosophical Quarterly.

